# Insulin sensitivity depends on the route of glucose administration

**DOI:** 10.1007/s00125-020-05157-w

**Published:** 2020-05-08

**Authors:** Geltrude Mingrone, Simona Panunzi, Andrea De Gaetano, Sofie Ahlin, Valerio Spuntarelli, Isabel Bondia-Pons, Chiara Barbieri, Esmeralda Capristo, Amalia Gastaldelli, John J. Nolan

**Affiliations:** 1 Fondazione Policlinico Universitario A. Gemelli IRCCS, Rome, Italy; 2 Università Cattolica del Sacro Cuore, Rome, Italy; 3 Division of Diabetes & Nutritional Sciences, Faculty of Life Sciences & Medicine, King’s College London, Denmark Hill Campus, 125 Coldharbour Road, London, SE5 9NU UK; 4 Steno Diabetes Center, Gentofte, Denmark; 5 CNR-IASI BioMatLab, Consiglio Nazionale delle Ricerche, Istituto di Analisi dei Sistemi ed Informatica, Laboratorio di Biomatematica (Italian National Research Council, Institute for System Analysis and Computer Science, Biomathematics Laboratory), Rome, Italy; 6 Department of Molecular and Clinical Medicine, Institute of Medicine, the Sahlgrenska Academy at the University of Gothenburg, Gothenburg, Sweden; 7 Cardiometabolic Risk Laboratory, Institute of Clinical Physiology, CNR, Pisa, Italy; 8 School of Medicine, Trinity College Dublin, Dublin, Ireland

**Keywords:** Insulin secretion, Insulin sensitivity, Isoglycaemia glucose infusion, Metabolomics, Stable isotopes

## Abstract

**Aims/hypothesis:**

The small intestine plays an important role in hepatic and whole-body insulin sensitivity, as shown by bariatric surgery. Our goal was to study whether routes and dose of glucose administration have an acute impact on insulin sensitivity. The primary endpoint of this proof-of-concept study was the difference in insulin-mediated metabolic clearance rate (MCR/I) of glucose between the oral and intravenous routes of glucose administration. Secondary endpoints were differences in insulin effect on proteolysis, ketogenesis, lipolysis and glucagon levels.

**Methods:**

In this parallel cohort study, we administered multiple oral glucose loads to 23 participants (aged between 18 and 65 years) with morbid obesity and with normal or impaired glucose tolerance or type 2 diabetes. In a different session, we administered isoglycaemic intravenous glucose infusions (IGIVI) to match the plasma glucose levels observed during the oral challenges. Glucose rate of appearance (*R*_a_) and disappearance (*R*_d_) and endogenous glucose production (EGP) were calculated by infusing [6,6-^2^H_2_]glucose with or without oral [U-^13^C_6_]glucose. Plasma small polar metabolites were measured by gas chromatography and time-of-flight mass spectrometry. Lipids were measured by ultra-HPLC and quadrupole mass spectrometry. Glucagon-like peptide-1, insulin, C-peptide and glucagon were also measured. Participants, caregivers, people doing measurements or examinations, and people assessing the outcomes were unblinded to group assignment.

**Results:**

Glucose MCR/I was significantly higher during IGIVI than during oral glucose administration, independently of glycaemic status (12 ± 6 for IGIVI vs 7.4 ± 3 ml min^−1^ kg^−1^ per nmol/l for oral, *p*< 0.001 from paired *t* test). Insulin secretion was higher during oral administration than during IGIVI (p< 0.001). The disposition index was significantly lower during the oral procedure: 4260 ± 1820 vs 5000 ± 2360 (ml min^−1^ kg^−1^ (nmol/l)^−1^ pmol/min; *p* = 0.005). Insulin clearance was significantly higher when glucose was infused rather than ingested (2.53 ± 0.82 vs 2.16 ± 0.49 l/min in intravenous and oral procedure, respectively, *p* = 0.006). The efficacy of insulin in inhibiting lipolysis and proteolysis was decreased after oral glucose loads. A heat map diagram showed a different pattern for the metabolites between the two routes of glucose administration.

**Conclusions/interpretation:**

Our study shows that insulin sensitivity depends on the route of glucose administration, the oral route leading to increased insulin secretion and compensatory insulin resistance compared with the intravenous route. The efficacy of insulin in blocking lipolysis and protein breakdown is lower after oral glucose loads vs the intravenous route. Our findings suggest that, while the glucose-mediated incretin release is followed by an increase in insulin release, the effect of the released insulin is limited by an increase in insulin resistance.

**Trial registration:**

ClinicalTrials.gov NCT03223129.

**Figure:**
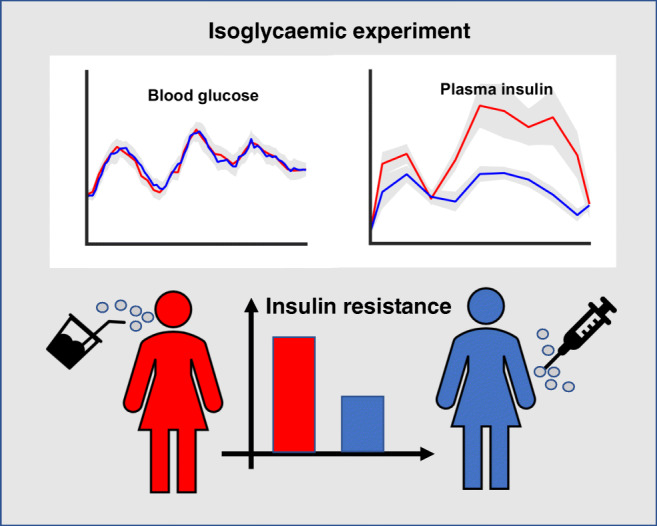
Graphical abstract

**Electronic supplementary material:**

The online version of this article (10.1007/s00125-020-05157-w) contains contains peer-reviewed but unedited supplementary supplementary material, which is available to authorised users.

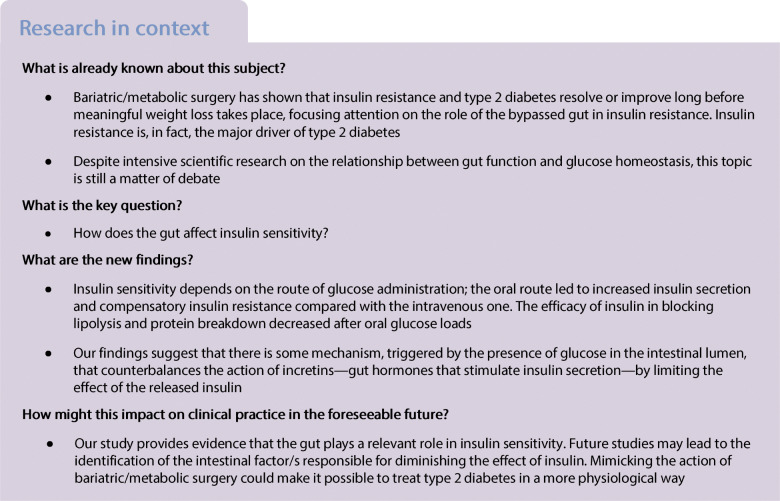



## Introduction

Until a few decades ago, diabetes was considered a disease essentially driven by pancreatic beta cell failure. While this concept holds true for type 1 diabetes, in type 2 diabetes insulin resistance develops long before beta cell failure and overt hyperglycaemia [1–3] and, thus, it is now recognised as the primary defect leading to type 2 diabetes.

Bariatric surgery has demonstrated the central role played by the small intestine in insulin resistance. It is of note that type 2 diabetes remission and insulin resistance reversal [4–10] following proximal gut bypass, such as in bilio-pancreatic diversion (BPD) and Roux-en-Y gastric bypass (RYGB), occur within a few days after the operation, when body weight is not yet significantly reduced [11, 12]. Despite intensive scientific research on the relationship between gut function and glucose homeostasis [13–15], this topic is still a matter of debate.

In the 1960s, it was shown that oral administration of 20 g of glucose in normal adults resulted in a much larger rise of plasma insulin compared with the administration of the same amount of glucose intravenously [16]. This ‘incretin effect’ was thereafter shown to depend on the secretion of glucagon-like peptide-1 (GLP-1) and glucose-dependent insulinotropic peptide (GIP), which stimulate insulin secretion [17]. Already in the first publication [16], it was observed that, notwithstanding higher plasma insulin concentrations, glucose clearance rates were similar after oral and intravenous glucose administration. It therefore seems likely that some form of suppression of insulin activity is at play after oral glucose dosing.

We hypothesised that a high flow of carbohydrates through the duodenum and upper jejunum, eliciting a high insulin secretory response, may also induce insulin resistance as a protective mechanism against hypoglycaemia. This mechanism is consistent with the ‘foregut hypothesis’, which holds that surgical exclusion of the proximal gut reduces intestinal factor/s that impair the action of insulin [18, 19].

The present study aims at making a significant step forward following on from our previous investigation [20]. Using a mathematical model, the previous study examined the impact of oral, as opposed to intravenous, glucose administration while holding insulin sensitivity constant. In this case, the subject would undergo hypoglycaemia [20]. That study [20] estimated the glucose absorption rate (rate of appearance; *R*_a_) of a single 75 g dose of glucose, while the present study measures it directly using stable isotopes and with increasing doses of glucose. To evaluate whether different degrees of insulin sensitivity coupled with a different insulin secretory efficiency could elicit distinct responses to the oral vs the intravenous route of glucose administration, we extended our previous investigation to different glycaemic states. Therefore, we enrolled individuals with normal glucose tolerance (NGT), with impaired glucose tolerance (IGT), which is considered to be a transition state between NGT and diabetes, and with type 2 diabetes. In addition, metabolomics was performed in order to investigate the effects of insulin in suppressing proteolysis, ketogenesis, lipolysis and glucagon levels.

## Methods

### Participants

The primary endpoint of this proof-of-concept study was the difference in insulin-mediated glucose metabolic clearance rate (MCR/I) between the oral and intravenous method of glucose administration. Secondary endpoints were differences in the insulin effect on proteolysis, ketogenesis, lipolysis and glucagon levels.

The sample size was calculated according to the MCR/I data reported in Gastaldelli et al. [21]. Assuming a 30% higher glucose MCR/I during isoglycaemic intravenous glucose infusion (IGIVI) (4.6 for oral vs 5.98 ml min^−1^ kg^−1^ per nmol/l for IGIVI, each SD = 0.9), α = 0.05 and power = 0.90, 24 participants (eight with NGT, eight with IGT and eight with type 2 diabetes) were required, using a more conservative *t* test for independent samples and considering a 25% attrition rate.

Inclusion criteria were: normal glucose tolerance or impaired glucose tolerance or type 2 diabetes; BMI> 30 kg/m^2^; age between 18 and 65 years; both sexes; and capacity to give informed consent. Exclusion criteria were: liver, kidney, cardiac or respiratory failure; major endocrine diseases requiring treatment; active cancer (surgical or medical treatment in the 5 years preceding the enrolment); HbA_1c_ ≥ 10% (85.5 mmol/mol) for participants with type 2 diabetes. Participants, caregivers, people doing measurements or examinations, and people assessing the outcomes were unblinded to group assignment.

The study was conducted at the University Hospital Policlinico Gemelli at Rome, Italy between July 2017 and July 2019. One participant initially allocated to the IGT group had NGT after re-examining the OGTT results and one participant with type 2 diabetes refused to undergo the intravenous study, and thus was excluded from the study. Therefore, nine participants with NGT, seven with IGT and seven with type 2 diabetes underwent oral and, after 7–10 days, intravenous glucose tests following a 12 h overnight fast on each occasion. Diabetes duration was 2–4 years and all patients were receiving oral hypoglycaemic agents (metformin alone or plus sodium–glucose cotransporter 2 inhibitors), which were discontinued 24 h before the studies.

The protocol was approved by the ethics committee of Catholic University of Rome, Italy. All participants provided written informed consent. Details on inclusion and exclusion criteria are reported in the ESM Methods.

### Biochemical measurements

To collect arterialised venous blood, a retrograde catheter was inserted in a dorsal hand vein, with the hand kept in a warming blanket. A forearm vein of the contralateral arm was catheterised for the infusions.

During the first session, at 08:00 h, [6,6-^2^H_2_]glucose was infused (priming: 22 μmol/kg; infusion rate: 0.22 μmol kg^−1^ min^−1^) to determine glucose kinetics. After 2.5 h of isotope infusion (basal period), an OGTT was given and consumed over 5 min. The OGTTs consisted of a 25 g solution followed by 75 g after 2 h and by 100 g after a further 2 h. Each OGTT contained 0.9 g of [U-^13^C_6_]glucose tracer. Plasma glucose was measured at baseline and every 10 min thereafter until 360 min.

In a different session, at 08:00 h, the participants were infused with a 20% wt/vol. adjustable glucose infusion in order to match the plasma glucose concentrations obtained during the OGTTs. After baseline blood samples were obtained, [6,6-^2^H_2_]glucose (22 μmol/kg prime and 0.22 μmol kg^−1^ min^−1^ constant infusion) was infused. At 10:30 h, after the basal period was completed, 20% dextrose enriched to approximately 2.5% with [6,6-^2^H_2_]glucose to minimise changes in glucose isotopic enrichment, was infused. Plasma glucose was measured every 10 min until 360 min, in order to change the glucose infusion rate to obtain an isoglycaemic pattern.

Plasma insulin, C-peptide, glucagon and GLP-1, as well as metabolites, were measured during fasting and, thereafter, every 20 min up to 360 min after starting the OGTT or the intravenous isoglycaemic infusion.

We will use the terms Time 1, Time 2 and Time 3 throughout the manuscript to indicate the different sub-experiments with increasing oral glucose loads (25, 75 and 100 g) and intravenous glucose infusion time periods performed to mimic the glycaemic response to the oral glucose challenges.

### Assays

Plasma glucose concentrations were determined by a glucose oxidase method using a glucose analyser. Insulin and C-peptide were measured by the Architect 1000 SR (Abbott Diagnostics, Abbott Park, IL, USA). Glucagon and total GLP-1 were measured by ELISA (Mercodia, Uppsala, Sweden).

### GC/MS analyses of glucose

Isotopic enrichment of [6,6-^2^H_2_]-glucose and [U-^13^C_6_]glucose was measured by electron impact ionisation on a GC/MS 5975 (Agilent Technologies, USA) using a 30 m× 0.25 mm HP-5MS column by monitoring ions at *m*/*z* 202/200 and 205/200, as previously described [22]. For all GC/MS analyses, instrument response was calibrated using standards of known enrichment. Glucose enrichment was good; the lowest average (mean) tracer-to-tracee ratio was 1%.

### Metabolomic platforms analysis

Plasma samples were analysed by two global profiling analytical platforms and a targeted profiling platform. Two-dimensional gas chromatography coupled with time-of-flight mass spectrometry (GC × GC-TOFMS) was applied to measure small polar metabolites [23]; and ultra-HPLC coupled with quadrupole mass spectrometry (UHPLC-QTOFMS) global lipid profiling was used to measure lipids [24, 25].

### Stable isotope calculations

Glucose *R*_a_ and rate of disappearance (*R*_d_) were calculated from changes in glucose enrichment according to the non-steady-state Steele equation and a time-varying glucose distribution volume to reduce the size of the non-steady-state error [26]. During the OGTTs, total glucose *R*_a_ was calculated by [6,6-^2^H_2_]glucose tracer/tracee ratio and the oral *R*_a_ by [U-^13^C_6_]glucose tracer/tracee [26]. The endogenous glucose production (EGP) was computed as (total *R*_a_ − oral *R*_a_).

### Statistics

#### Computation of indices

Data from the OGTTs were analysed by the oral glucose minimal-model [27]. This model was adapted to estimate quantities of interest using all data points from the three sequential OGTTs. In particular, for each participant, static, dynamic and global beta cell sensitivity (ϕ_s_, ϕ_d_ and ϕ_global_, respectively) during the three time periods were computed through equation numbers 8, 9 and 11 from this study [27] by including in the model three different β parameters and three different *K*_*d*_ constants and by estimating them in a single optimisation procedure.

Insulin sensitivity index in the three time intervals could not be derived according to Breda et al. [27] since the AUCs in the equations used must ideally be calculated from 0 to ∞, when blood glucose returns to baseline. For this reason, we calculated the glucose MCR/I as (*R*_d_/plasma glucose)/plasma insulin (calculated as ml min^−1^ kg^−1^ per pmol/l, and multiplied by 1000 to transform it into ml min^−1^ kg^−1^ body weight per nmol/l) over the entire experiment, and average AUCs, computed separately on the three time intervals and per study group, were calculated to estimate insulin sensitivity [28].

The product of EGP and plasma insulin concentration was used as an index of hepatic insulin resistance. The insulinogenic index was computed as the ratio between incremental insulin AUC and incremental glucose AUC. Insulin secretion rate (ISR) was computed using the two-compartment model for C-peptide distribution and degradation as described by Van Cauter et al. [29]. Insulin clearance was computed according to the formula ISR-AUC/insulin-AUC – *V*×(insulin at final time – insulin at initial time)/insulin-AUC, as proposed by Jung et al. [30], where *V* represents the insulin distribution volume set to 0.14 l/kg as reported in [30].

For each participant, disposition index (DI) was computed over time as the product of ISR and MCR/I and then averaged. The adipose tissue insulin resistance (Adipo-IR) is a way to measure the resistance to the anti-lipolytic effect of insulin. Adipo-IR [31] was computed as the sum of mean fatty acid levels multiplied by the mean circulating insulin levels.

The disappearance rates of all amino acids, isoleucine and lactic acid by glycaemic group and by experimental procedure were estimated by a decaying exponential model. Insulin normalised beta values were also computed by dividing per mean insulin levels.

#### Statistical analysis

Differences between study groups (inside each experimental procedure or independently of experimental procedure) were evaluated by ANOVA; differences between experimental procedures (independently of study groups) were evaluated by paired *t* test or Wilcoxon test.

Mixed-effects models were used to evaluate variations over time of continuous variables, with experimental procedure as within-factor and glycaemic group as between-factor. A two-sided *p*< 0.05 was considered significant.

Each metabolite was analysed by a mixed-effects model including time (Time 1, Time 2 and Time 3), procedure and glycaemic group as predictors. For each metabolite, percentage Δ (difference between final and baseline concentrations over baseline), were used to perform both a partial least-squares discriminant analysis (PLS*-*DA*)* and a random forest analysis in order to discriminate experiments (oral vs intravenous) on the basis of classes of metabolite variation.

Heat maps were used as a graphical representation of metabolomic mean values per categories of subjects during the three time periods (Time 1, Time 2 and Time 3) and the two experimental procedures, including only metabolites for which the procedure and/or time × experimental procedure interaction from the mixed-effects analyses were significant. All statistical analyses were conducted in R [32].

## Results

The study participants were of similar age (*p* = 0.65) and BMI (*p* = 0.48) included nine with NGT (46.0 ± 8.9 years, 53.7 ± 10.2 kg/m^2^), seven with IGT (41.1 ± 15.5 years, 49.1 ± 4.11 kg/m^2^) and seven with type 2 diabetes (46.7 ± 12.8 years, 53.0 ± 6.7 kg/m^2^). Variables measured at baseline are reported in Table 1.

**Table 1:** Anthropometric characteristics and metabolic and biochemical measurements of the participants

	NGT (*n* = 9)		IGT (*n* = 7)		Type 2 diabetes (*n* = 7)		Total (*N* = 23)		
Variables	Mean	SD	Mean	SD	Mean	SD	Mean	SD	P
Age (years)	46.0	8.89	41.14	15.52	46.71	12.76	44.74	12.03	0.655
Weight (kg)	152.44	24.39	135.29	24.8	153.57	26.89	147.57	25.51	0.325
BMI (kg/m^2^)	53.71	10.17	49.1	4.11	52.97	6.69	52.08	7.66	0.478
Plasma glucose (mmol/l)	5.35	0.61	4.905	0.37	7.10	3.42	5.75	2.06	0.0055
2 h Glucose (mmol/l)	6.06	0.75	5.7	1.11	7.53	1.40	6.41	1.30	0.012
HbA_1c_ (mmol/mol)	37.11	3.41	37.14	2.61	61.43	24.42	44.52	17.3	0.003
HbA_1c_ (%)	5.55	2.46	5.55	2.39	7.77	4.38	6.22	3.73	
BUN (mmol/l)	5.32	0.63	4.90	0.49	4.95	0.84	5.08	0.66	0.395
Creatinine (mmol/l)	75.83	10.41	75.39	15.79	63.77	16.81	72.03	14.69	0.21
Uric acid (mmol/l)	0.37	0.06	0.38	0.08	0.40	0.07	0.38	0.07	0.67
Total cholesterol (mmol/l)	4.61	0.37	4.91	0.47	5.04	1.23	4.84	0.75	0.516
HDL-cholesterol (mmol/l)	1.27	0.17	1.38	0.29	1.18	0.29	1.28	0.25	0.371
LDL-cholesterol (mmol/l)	2.65	0.36	2.70	0.35	3.23	1.12	2.84	0.70	0.22
Triacylglycerol (mmol/l)	1.06	0.22	1.54	0.64	1.45	0.35	1.32	0.46	0.076

BUN, blood urea nitrogen

### Primary outcome

**Fig. 1:**
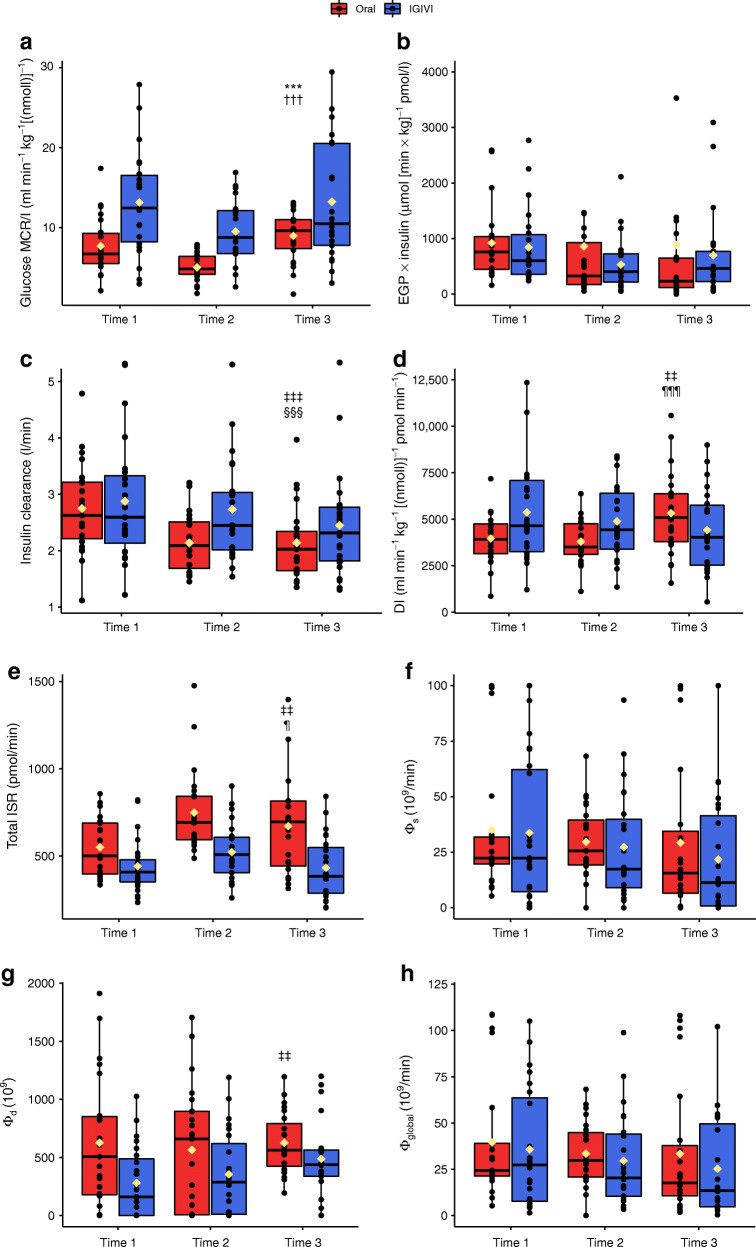
(**a**) Whole-body insulin sensitivity measured with stable isotopes as MCR/I, i.e. *R*_d_ from general circulation normalised by circulating levels of glucose and insulin. (**b**) EGP measured by stable isotopes and multiplied by circulating levels of insulin was used as an index of hepatic insulin resistance. (**c**) Insulin clearance. (**d**) DI and (**e**) the total ISR were calculated as detailed in the Methods. (**f**) Static (Φ_s_), (**g**) dynamic (Φ_d_) and (**h**) global (Φ_global_) beta cell glucose sensitivity (which results from the sum of the two components above). Data are shown in relation to the three time periods (Time 1, Time 2 and Time 3). Black circles represent single participant values whereas yellow diamonds represent mean values. ***p< 0.001 for paired *t* test comparing oral vs IGIVI administration (average over the three time points); ^†††^*p*< 0.001 for β coefficient for Time 2 vs Time 1; ^‡‡^*p*< 0.01, ^‡‡‡^*p*< 0.001 for experiment factor significance; ^§§§^*p*< 0.001 for time factor significance; ^¶^*p*< 0.05 ^¶¶¶^*p*< 0.001 for experiment/time interaction term significance

The insulin-mediated glucose MCR/I (primary endpoint) was significantly higher during IGIVI than during oral (OGTT) glucose administration, independent of glycaemic status (12 ± 6 for IGIVI vs 7.4 ± 3 ml min^−1^ kg^−1^ per nmol/l for oral [average over the three time points], *p*< 0.001 from paired *t* test). Glucose MCR/I significantly decreased during the second sub-experiment (Time 2) (β = −0.003, SE = 0.0008, p< 0.001), while increasing again with the highest dose of glucose (Time 3) (Fig. 1a).

### Glucose and insulin profiles

**Fig. 2:**
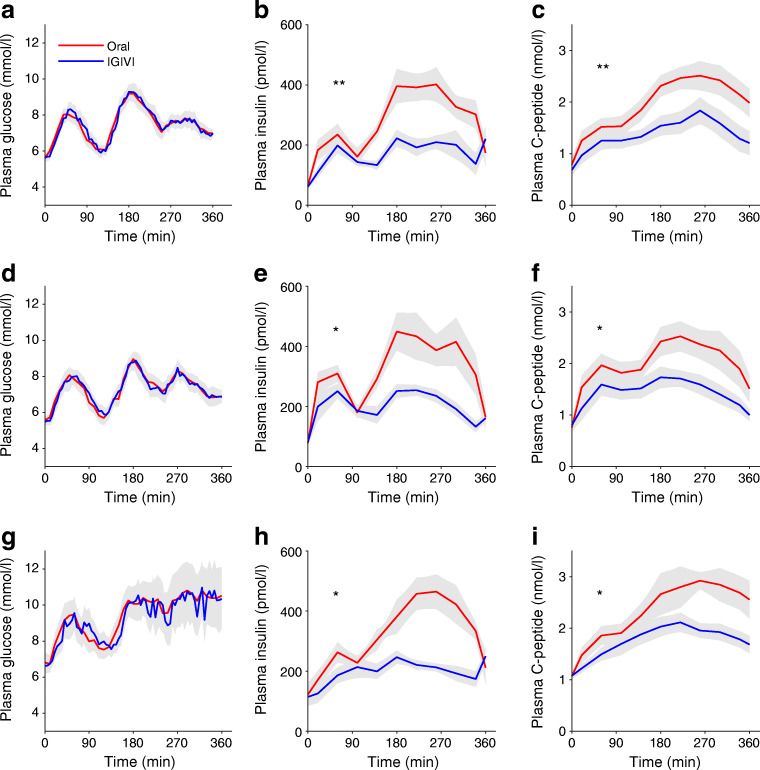
Time courses of (**a**, **d**, **g**) plasma glucose, (**b**, **e**, **h**) insulin and (**c**, **f**, **i**) C-peptide levels during the OGTT (oral) or the isoglycaemic intravenous (IGIVI) glucose administration in the three groups of participants: participants with NGT (**a**, **b**, **c**), IGT (**d**, **e**, **f**) and type 2 diabetes (**g**, **h**, **i**). *p< 0.05 and **p< 0.01 by Wilcoxon test comparing the supra-basal insulin AUC and the C-peptide AUC during the oral and the IGIVI glucose administration for each of the three groups of participants

Isoglycaemia was reached in all groups (Fig. 2). For each study group, glucose incremental AUCs were computed and tested for differences between experimental procedures by Wilcoxon tests. None of the tests was significant (*p* = 0.82 for NGT, *p* = 0.58 for IGT and *p* = 0.81 for type 2 diabetes). A higher peak plasma glucose was observed in type 2 diabetes than in NGT and IGT (NGT 9.79 ± 1.01; IGT 9.61 ± 1.32; type 2 diabetes 12.38 ± 3.62 mmol/l; *p* = 0.047).

Insulin concentrations were significantly (*p*< 0.001) higher during the oral test than during IGIVI (Fig. 2), but did not differ significantly among groups. The incremental insulin AUC was higher during the oral test (81,706.1 ± 37,438.7 pmol/l × min) than during the IGIVI procedure (38,538.9 ± 14,813.0 pmol/l × min; *p* = 0.012 from mixed-effects model) with no significant differences among groups.

### Glucagon and GLP-1

**Fig. 3:**
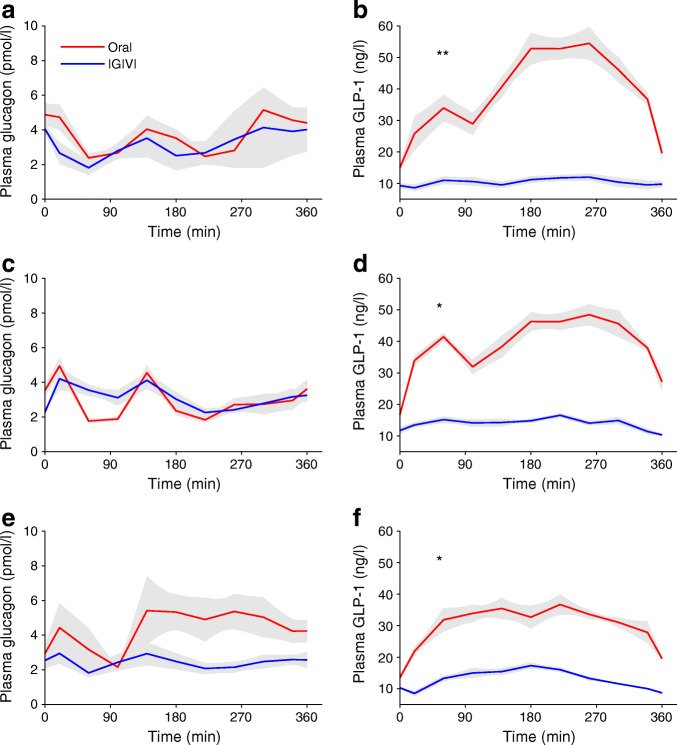
Time courses of (**a**, **c**, **e**) plasma glucagon and (**b**, **d**, **f**) GLP-1 levels during the OGTT (oral) or the isoglycaemic intravenous (IGIVI) glucose administration in the three groups of participants: participants with NGT (**a**, **b**), IGT (**c**, **d**) and type 2 diabetes (**e**, **f**). *p< 0.05 and **p< 0.01 by Wilcoxon test comparing the glucagon and GLP-1 AUCs during the oral and the IGIVI glucose administration for each of the three groups of participants

No differences were observed when comparing AUCs or mean levels of glucagon (Fig. 3) between experiments and among groups. GLP-1 concentrations were significantly higher during oral administration than during IGIVI (mean GLP-1-AUC: 13557.1 ± 2748.3 vs 4509.8 ± 1010.4 ng/l *×* min for oral and intravenous routes, respectively; mean GLP-1: 37.4 ± 7.6 vs 12.5 ± 2.8 ng/l, *p*< 0.001), with type 2 diabetic participants secreting less GLP-1 than participants with NGT during oral glucose administration (*p* = 0.0012). Interestingly, GLP-1 secretion failed to inhibit glucagon secretion after oral glucose in participants with type 2 diabetes (*p* = 0.0035).

### Insulin sensitivity and OGTT modelling

Glucose MCR/I was not significantly different among glycaemic groups (ESM Fig. 1a). The insulinogenic index was significantly different in the two experimental procedures: 149.0 ± 122.1 vs 70.7 ± 60.6 in OGTT and IGIVI, respectively (*p*< 0.0001). No differences among glycaemic groups were observed (data not shown in the figures).

Oral glucose administration reduced hepatic insulin sensitivity by impairing insulin-mediated suppression of EGP (888.9 ± 1347.9 in oral vs 691.4 ± 668.7 μmol [min *×* kg]^−1^ pmol/l in IGIVI [average over the three time points], *p* = 0.056 from paired *t* test), although the significance was borderline. The index did not differ in the three time periods (Time 1, Time 2 and Time 3) (Fig. 1b).

Insulin clearance was significantly higher when glucose was infused rather than ingested (2.53 ± 0.82 vs 2.15 ± 0.49 l/min in IGIVI and oral, respectively [average over the three time points], *p* = 0.006). Moreover, it decreased during the three time periods (Time 1, Time 2 and Time 3) (*p*< 0.001 for time factor), except for Time 2 of the IGIVI procedure (*p* = 0.037) (Fig. 1c).

The DI was significantly lower during the oral procedure (4260 ± 1820 vs 5000 ± 2360 ml min^−1^ kg^−1^ [nmol/l]^−1^ pmol min^−1^ [average over the three time points], *p* = 0.005). The interaction term was also significant (*p* = 0.0008): during the third sub-experiment DI increased during the oral procedure while it decreased during the IGIVI (Fig. 1d).

Total ISR was higher during the oral administration than during IGIVI (656.7 ± 239.9 vs 465.6 ± 168.0 pmol/min, respectively [average over the three time points], *p*< 0.001). Total ISR increases significantly during the Time 2 and Time 3 during the oral procedure, while in the IGIVI it decreases during the third experiment (p< 0.001 for time and *p* = 0.023 for time × procedure) (Fig. 1e).

Among the indices of beta cell glucose sensitivity, only the dynamic beta cell glucose sensitivity ϕ_d_ was significant, with the experiment (oral or IGIVI) being the only significant factor (*p* = 0.004) and the estimated marginal means being 612.77 (SE = 59.81) for the oral and 369.68 (SE = 47.18) for the IGIVI.

A mixed-effects model was used to test a linear relationship between insulin secretion (average AUC-ISR) and MCR/I index, computed in the three time periods (Time 1, Time 2 and Time 3; increasing dose of oral glucose, from 25 g to 100 g), considering the procedure (oral vs IGIVI) as an additional predictor. Both regression coefficients were negative and significant, showing that insulin secretion decreases while insulin sensitivity increases (*p*< 0.0001) and that this behaviour is different, depending on the route of glucose administration (p< 0.0001).

Differences in the metabolic parameters among glycaemic groups are reported in ESM Table 1.

### Metabolomic data analysis

#### PLS-DA analysis, heat map and random forest plot

**Fig. 4:**
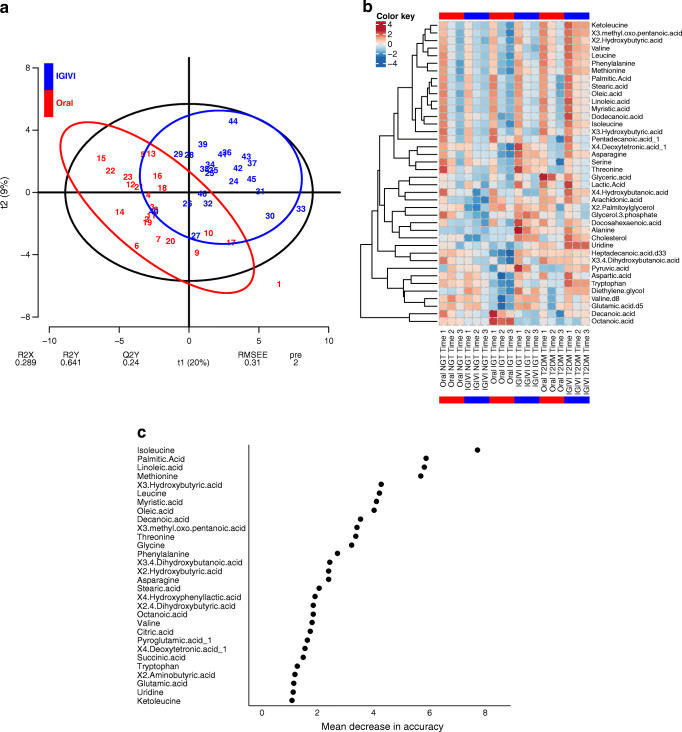
(**a**) PLS-DA score plot used to discriminate between OGTT (oral) and intravenous (IGIVI) experiments on the basis of classes of metabolite variation during the experimental procedures. Numbers refers to participant labels, red for scores during oral and blue for scores during IGIVI glucose administration. (**b**) Heat map of significant procedure-associated metabolite changes (oral vs IGIVI) per category of participant (NGT, IGT and type 2 diabetes [T2DM]) and experiment time (3 time periods, Time 1, Time 2 and Time 3). Blue bars group values of IGIVI experiments and red bars group values of oral experiment. The colour key shows the standard *z* score values. (**c**) Importance plot (mean decrease in accuracy) from random forest analysis. Pre, number of selected components; Q2Y, fraction of the variation of the *y* variables predicted by the model; R2X, fraction of the variation of the *x* variables (predictors) explained by the model; R2Y, fraction of the variation of the *y* variables (response) explained by the model; RMSEE, root-mean-square error of estimation values; t1 and t2, axes of the score plot (with the percentage of variation of the X variables explained by each component in parentheses)

A PLS-DA explained about 30% of the global variability of metabolite levels (Fig. 4a) and showed a good classification performance (misclassification error rate = 0.087). The first component correlated positively with the intravenous (correlation coefficient 0.66) and negatively with the oral experiments (−0.66). X3.Hydroxybutyric acid, isoleucine, myristic acid, palmitic acid, oleic acid, stearic acid, leucine, linoleic acid and threonine correlated positively with the first component (>0.80), meaning that the route of glucose administration determined different decrements of these metabolites.

Changes in metabolites by times and glucose tolerance states (NGT, IGT, type 2 diabetes) are presented in a clustered heat map (Fig. 4b). In the heat map the rows represent single metabolites and the columns represent the oral or intravenous glucose challenges at the three time points of increasing doses (indicated as Time 1, Time 2 and Time 3) in participants with NGT, IGT and type 2 diabetes. Highly decreased metabolites are displayed in blue, while highly increased metabolites are displayed in red. The intensity of each colour corresponds to the magnitude of the difference when compared with the average value.

As expected, increased glucose loads were associated with lower levels of fatty acids and amino acids by increasing insulin secretion. This effect was more pronounced for participants with NGT and IGT than for those with type 2 diabetes. The circulating levels of docosahexaenoic and dodecanoic acids increased more during intravenous than during oral glucose administration.

To assess the ability to classify participants in relation to the route of glucose administration (oral vs intravenous), a random forest analysis was performed. The random forest model identified seven metabolites (isoleucine, methionine, linoleic acid, palmitic acid, X3.hydroxybuyiric acid, myristic acid and oleic acid) as key in classifying the data. The analysis shows excellent accuracy in predicting the group (oral vs IGIVI), with 84.8% participants from each group identified correctly and 15.2% out of bag error rate. Figure 4c reports the importance variable plot.

#### Effect of increasing doses of glucose

The levels of circulating metabolites were related to the time (dependent on the three doses of glucose used), the type of experiment (oral vs IGIVI) and the glycaemic status. The β coefficients of metabolites and their *p* values from the mixed-effects model with experiment, time and the relative interaction as within-factor and glycaemic status as between-factor are reported in ESM Table 2. β coefficients of metabolites with their *p* values from the mixed-effects model multiplied by insulin with experiment, time and the relative interaction as within-factor and glycaemic status as between-factor are reported in ESM Table 3. The levels of these metabolites were lower at Time 3 than during Time 1, but decreased less during IGIVI. In contrast, pyruvic acid decreased most during Time 3 of the IGIVI.

Multiplying the metabolite levels by the insulin level (ESM Table 3), the metabolite concentrations increased over time during both methods of glucose administration but they were always lower during IGIVI rather than during oral administration.

#### Adipo-IR and metabolite *R*_d_

**Fig. 5:**
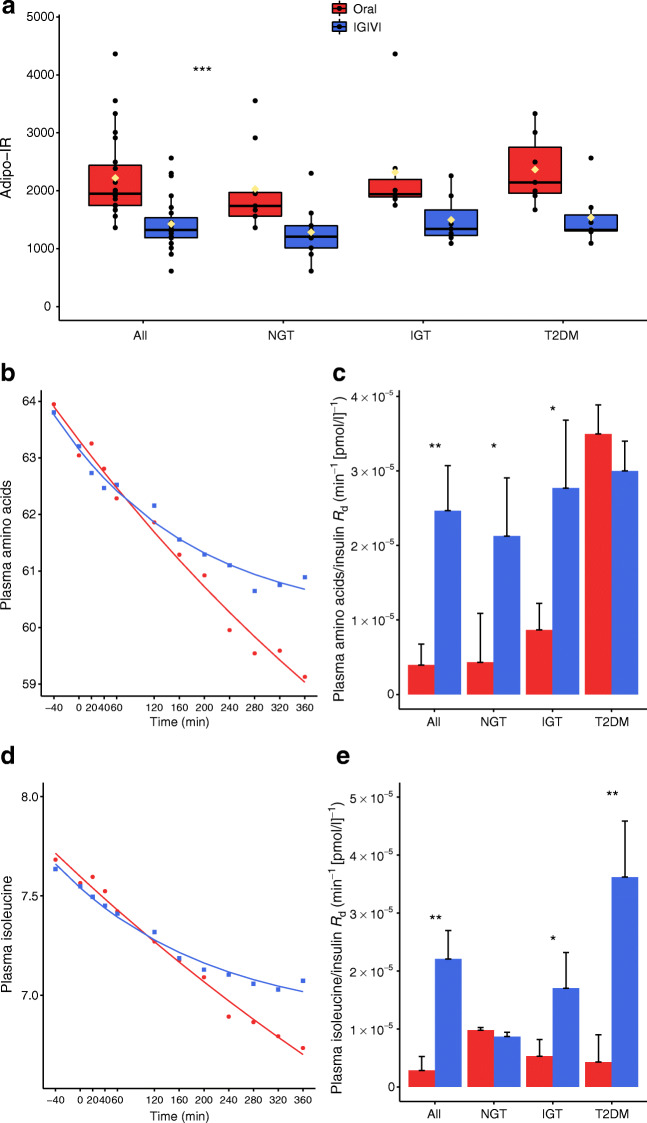
(**a**) Adipo-IR index obtained by multiplying the levels of fatty acids by the average insulin levels during the two procedures (OGTT [oral] vs IGIVI). Black circles represent single participant values whereas yellow diamonds represent mean values. Significant experiment effect from mixed effects model. (**b**, **d**) The capacity of insulin to clear combined amino acids (**b**) or isoleucine (**d**) from the circulatory stream was estimated by adopting a decaying exponential model (points represent observed values while continuous lines represent model predictions). (**c**, **e**) The bar graphs show the values of the estimated *R*_d_ normalised by insulin per experimental procedure and glycaemic group. Comparisons between R_d_ coefficients in each group are from *Z* tests. *p< 0.05, **p< 0.01, ***p< 0.001 are the test significance for oral vs IGIVI

Figure 5a shows the Adipo-IR index by experimental procedure and by glycaemic group. Figure 5b,d shows the time course of amino acids and isoleucine during oral administration vs IGIVI and the relative predictions from a decaying exponential model. Figure 5c,e shows the estimated rates of decay *R*_d_ (exponential coefficient β) per unit of insulin among the various glycaemic groups. The raw *R*_d_ was significant for combined amino acids and isoleucine (*p* = 0.0068 and *p* = 0.002, respectively). The raw *R*_d_ value for lactic acid was 0.0009 ± 0.0003 1/min for oral administration and 0.0014 ± 0.00016 1/min for IGIVI (*p* = 0.066). If normalised by mean insulin levels the difference was highly significant.

## Discussion

Our study focuses on the differential effect of different routes of glucose administration, oral vs intravenous, on insulin sensitivity. We found that insulin sensitivity was significantly lower when glucose was taken orally rather than given intravenously. This effect was independent of the participant’s glycaemic status, although individuals with type 2 diabetes showed a higher insulin resistance when compared with individuals with IGT or NGT. Multiple and increasing glucose loads enhanced glucose disposal.

Not only was peripheral insulin sensitivity, expressed as glucose MCR/I, blunted during the oral vs the intravenous route of glucose administration, but hepatic insulin clearance was also reduced. This latter is, in fact, a typical feature of hepatic insulin resistance [33].

Our results confirm the observations of Nauck et al. [34], who showed a significantly reduced fractional hepatic insulin extraction after oral glucose administration (46.9–54.6%) compared with an IGIVI (63.4–76.5%), suggesting higher hepatic insulin resistance when glucose was given orally. However, Nauck et al. [34] did not use stable isotopes.

The ‘incretin effect’ is the phenomenon by which the same plasma glucose concentration elicits a much higher insulin secretion during oral rather than intravenous glucose administration [34]. It is, however, unclear how an individual would not develop hypoglycaemia as a consequence of this higher insulin secretory response. The observation that, after oral glucose administration, the anti-lipolytic and anti-proteolytic action of insulin is blunted in the context of matching plasma glucose levels and similar glucose *R*_a_, and moreover that glucose MCR/I is reduced, points to the development of insulin resistance when glucose is given orally. In fact, should insulin act similarly after oral glucose loads, we would observe stronger anti-lipolytic and anti-proteolytic effects.

The DI, which reflects the ability to respond to insulin resistance by delivering more insulin into the peripheral circulation through increasing insulin secretion and/or reducing hepatic insulin clearance, was significantly lower during the oral procedure because when ISR increases, glucose MCR/I decreases as an adaptive mechanism. However, the DI increased after repeated oral glucose loads in agreement with the Staub–Traugott effect [35, 36], showing that repeated administrations of glucose facilitate glucose disposal [35–37].

Circulating GLP-1 levels progressively increased with increasing amounts of glucose ingested; this GLP-1 response was more pronounced in participants with NGT than in those with type 2 diabetes. Sjøberg et al. [38] demonstrated that at physiological levels GLP-1 does not affect whole-body insulin sensitivity. In fact, the GLP-1 analogue, exenatide, improves both hepatic and adipose insulin resistance but at plasma levels ten times higher than the GLP-1 levels elicited by an OGTT [22]. This suggests that the effects of the oral glucose challenge on insulin secretion and insulin sensitivity are mediated by two different players, one of which is already known, i.e. GLP-1, and the other not yet identified.

The secretion of glucagon was higher during oral glucose administration in participants with type 2 diabetes, suggesting insufficient glucagon secretion suppression by insulin and GLP-1 in these individuals.

Comparison of metabolites in the heat map showed a clear separation between metabolite profiles in relation to the mode of glucose administration. Metabolomics was performed in a non-targeted mode and, thus, statistical results are to be considered merely exploratory. As reported in ESM Table 2, a linear mixed-effects model showed that hydroxybutyric acid, branched chain amino acids (valine, leucine and isoleucine) and the leucine metabolite ketoleucine, as well as fatty acids (oleic, linoleic, palmitic, stearic and myristic acids) varied the most between the two modalities of glucose administration (interaction coefficient Time 3:IGIVI). The importance rank plot of metabolites (Fig. 4c) shows that most of the above metabolites are the most relevant ones in separating oral vs intravenous modalities of glucose administration, with a prediction accuracy of almost 85%.

Even though the circulating levels of insulin were doubled when glucose was given orally, they failed to suppress lipolysis, proteolysis or glucagon secretion, a crucial hormone for maintaining EGP [39, 40].

The Adipo-IR index [31] is considered to reflect adipose tissue resistance to the anti-lipolytic effects of insulin. We found that the Adipo-IR index was higher with oral glucose administration, suggesting impaired suppression of lipolysis in the presence of higher insulin levels when glucose was given orally.

Circulating levels of medium-chain fatty acids, docosahexaenoic and dodecanoic acids, increased more when increasing doses of glucose were given intravenously and may account for the higher insulin sensitivity observed during the intravenous infusion of glucose compared with glucose oral administration. Medium-chain fatty acids, in fact, exert beneficial effects on diabetes, obesity and inflammation [41], reduce body fat [42], enhance energy-expenditure [43] and prevent insulin resistance [43]. Similar to the effect on lipids, the efficacy of insulin in inhibiting proteolysis was decreased after oral glucose loads.

Taken together, these findings suggest that there is a mechanism, somehow triggered by the presence of glucose in the intestinal lumen, directed to counterbalancing incretin action by limiting the effect of the insulin released.

The oral boluses of glucose given in our study are consistent with the levels of glucose consumed in a typical meal by individuals with obesity who eat sweet bakery items and drink glucose-sweetened beverages. In fact, 25 g of glucose corresponds to one large soft drink. Two cake slices (200 g each) plus a small soft drink (250 ml) provide 75 g of glucose, while one and a half slices of a cake plus two large soft drinks deliver around 100 g of glucose.

Some limitations should be recognised in this study. Although the results are consistent, the sample size was limited and, by design, we did not use the euglycaemic−hyperinsulinaemic clamp, which is the gold standard for insulin sensitivity measurement. We also used multiple oral glucose loads to determine the dose–response in two single studies for participant convenience and in order to limit day-to-day variability. A potential bias derives from the fixed sequence of glucose administration, oral first and then intravenous, which was necessary according to the design of the study and use of chronic medications. Furthermore, our patients were affected by morbid obesity and it is not certain that our results can be extrapolated to individuals with lesser degrees of obesity. Finally, the Adipo-IR index is only a surrogate measure of NEFA turnover. Future research would permit the identification of the gut mediator/s of insulin resistance and possibly provide alternatives to surgery.

In conclusion, our study shows that the degree of insulin sensitivity depends on the route of glucose administration. Oral glucose administration leads to increased insulin secretion and compensatory insulin resistance compared with intravenous glucose administration. The MCR/I is significantly enhanced when glucose is administered intravenously rather than orally. EGP tends to increase (although not significantly, possibly due to high inter-individual variability), while insulin clearance is decreased when glucose is given orally rather than intravenously, in spite of similar rates of glucose appearance, pointing toward hepatic insulin resistance. Increased hepatic insulin resistance results in turn in increased circulating levels of gluconeogenetic metabolites. Our findings suggest that, while glucose-mediated incretin release is followed by an increase in insulin release, the effect of the released insulin is limited through an increase in insulin resistance.

## Electronic supplementary material

ESM 1 (PDF 310 kb)

## Data Availability

The datasets generated during the current study are available from the corresponding author on reasonable request.

## Abbreviations

Adipo-IR: Adipose tissue insulin resistance
DI: Disposition index
EGP: Endogenous glucose production
GLP-1: Glucagon-like peptide-1
IGIVI: Isoglycaemic intravenous glucose infusion
IGT: Impaired glucose tolerance
ISR: Insulin secretion rate
MCR/I: Insulin-mediated metabolic clearance rate
NGT: Normal glucose tolerance
PLS-DA: Partial least-squares discriminant analysis
*R*_a_: Rate of appearance
*R*_d_: Rate of disappearance
