# Supplementary material for: Insulin sensitivity depends on the route of glucose administration
Source: Diabetologia. 2020 May 8;63(7):1382–95. doi: 10.1007/s00125-020-05157-w (PMC7286868; doi:10.1007/s00125-020-05157-w)
Supplement: Supplementary file 1 — (PDF 310 kb) [file 125_2020_5157_MOESM1_ESM.pdf]

		NGT (n=9)		IGT (n=7)		T2DM (n=7)		Total (n=23)		
		**Mean**	**SD**	**Mean**	**SD**	**Mean**	**SD**	**Mean**	**SD**	***P**
**RoD [µmol/(min kg)]**	OGTT	15.7	2.2	16.0	2.9	13.9	3.8	15.2	3.0	0.37
	IGIVI	14.5	4.1	13.4	2.4	14.8	5.3	14.3	4.0	0.81
**EGP × I [µmol/(min kg) pM]**	OGTT	968.2	1668.8	771.8	616.5	891.6	745.9	885.1	1129.1	0.95
	IGIVI	883.9	812.2	519.8	235.3	1111.9	1163.7	842.5	825.4	0.42
**MCR/I** [l min^{-1} kg^{-1} per nmol/l]	OGTT	8.4	2.6	7.7	2.2	5.4	2.1	7.3	2.6	0.06
	IGIVI	14	5.2	11.3	5.8	10.3	4.2	12	5.1	0.33
**Insulin Clearance [l/min]**	OGTT	2.2	0.6	1.9	0.2	2.4	0.5	2.2	0.5	0.12
	IGIVI	2.5	0.9	2.1	0.4	3.0	0.8	2.6	0.8	0.10
**DI**	OGTT	4800	1700	4000	800	3900	1700	4300	1500	0.44
	IGIVI	5200	2000	4200	1800	5600	2200	5000	2000	0.40
**Total ISR [pmol/min]**	OGTT	220369.3	48889.4	219044.6	79632.3	278896.3	71258.2	237778.7	68971.3	0.17
	IGIVI	151710.3	53320.8	153822.1	55589.5	205185.7	45032.5	168628.2	55152.3	0.11
**Φ_S [10^9 min^{-1}]**	OGTT	26.5	17.3	35.9	20.3	33.0	19.4	31.3	18.5	0.60
	IGIVI	38.0	20.2	25.2	22.5	16.6	18.3	27.6	21.5	0.13
**Φ_D [10^{-9}]**	OGTT	532.8	303.3	654.6	245.2	650.8	296.0	605.8	278.1	0.62
	IGIVI	425.2	157.4	430.5	312.0	253.3	194.6	374.5	229.4	0.26
**Φ_{global} [10^9 min^{-1}]**	OGTT	29.9	18.3	40.2	20.7	37.7	19.3	35.4	19.0	0.55
	IGIVI	40.9	20.6	28.0	24.0	18.5	19.0	30.2	22.4	0.13

* P values from ANOVA

ESM Table 1 : RoD= rate of glucose disappearance; EGP=endogenous glucose production; MCR=metabolic clearance rate; DI=disposition index; ISR=insulin secretion rate, Φ_S =static beta cell sensitivity; Φ_D =dynamic beta cell sensitivity; Φ_{global} =global beta cell sensitivity

Metabolite	time2	P-value	time3	P-value	IGIVI	P-value	IGT	P-value	T2DM	P-value	time2:IGIVI	P-value	time3:IGIVI	P-value
X3.Hydroxybutyric.acid	-1.25	1.55E-18	-1.79	8.28E-29	-0.46	0.000	-0.049	0.798	0.454	0.027	0.325	0.053	0.642	0.000
Alanine	0.00	9.69E-01	-0.03	4.98E-01	0.20	0.000	0.199	0.040	0.193	0.046	-0.101	0.166	-0.138	0.060
Isoleucine	-0.46	2.10E-10	-0.71	3.38E-19	-0.04	0.590	-0.050	0.510	0.184	0.022	0.122	0.191	0.293	0.002
Leucine	-0.43	9.02E-11	-0.66	2.85E-19	-0.07	0.230	-0.096	0.163	0.273	0.001	0.118	0.169	0.255	0.003
Linoleic.acid	-0.66	2.79E-13	-0.96	1.20E-21	-0.05	0.507	0.014	0.897	0.246	0.036	0.144	0.203	0.307	0.008
Methionine	-0.36	1.18E-05	-0.53	6.12E-10	-0.03	0.719	0.021	0.821	0.242	0.017	0.211	0.059	0.260	0.020
Palmitic.Acid	-0.52	6.65E-13	-0.77	5.84E-22	-0.02	0.778	0.103	0.344	0.144	0.189	0.093	0.302	0.278	0.002
Phenylalanine	-0.24	6.59E-06	-0.38	2.30E-11	-0.02	0.651	0.014	0.887	0.160	0.124	0.074	0.308	0.177	0.016
Serine	-0.20	2.45E-02	-0.45	1.39E-06	-0.01	0.891	-0.035	0.682	-0.105	0.220	0.092	0.459	0.282	0.024
Stearic.acid	-0.39	2.24E-12	-0.58	8.12E-21	-0.04	0.428	0.038	0.635	0.095	0.242	0.100	0.157	0.198	0.006
Valine	-0.20	3.34E-09	-0.32	8.93E-18	-0.05	0.151	-0.047	0.326	0.177	0.001	0.042	0.351	0.115	0.011
Oleic.acid	-1.00	6.87E-14	-1.54	2.51E-24	-0.04	0.718	0.043	0.790	0.166	0.307	0.257	0.123	0.632	0.000
Tryptophan	-0.09	1.21E-01	-0.13	3.75E-02	0.21	0.001	-0.291	0.030	0.018	0.888	-0.050	0.551	-0.044	0.603
Lactic.Acid	-0.09	8.90E-02	-0.23	2.94E-05	0.16	0.004	0.023	0.748	0.156	0.039	-0.124	0.105	-0.161	0.036
Pyruvic.acid	0.17	4.51E-02	0.16	5.93E-02	0.31	0.000	0.058	0.638	-0.010	0.937	-0.216	0.065	-0.302	0.011
X2.Hydroxybutyric.acid	-0.37	9.26E-06	-0.77	2.82E-16	-0.04	0.594	0.065	0.715	0.370	0.049	0.059	0.605	0.230	0.046
X3.methyl.oxo.pentanoic.acid	-0.41	7.72E-07	-0.72	3.31E-15	-0.04	0.606	-0.024	0.862	0.399	0.009	0.176	0.116	0.381	0.001
Decanoic.acid	-0.17	3.20E-02	-0.29	2.83E-04	-0.19	0.016	0.028	0.754	-0.050	0.582	0.105	0.339	0.209	0.059
Diethylene.glycol	-0.06	1.67E-01	-0.03	4.91E-01	0.13	0.003	-0.024	0.727	0.008	0.905	0.011	0.862	-0.027	0.657
Dodecanoic.acid	-0.72	4.34E-09	-0.88	3.22E-12	-0.03	0.788	-0.065	0.669	0.172	0.263	0.325	0.043	0.333	0.038
Ketoleucine	-0.37	3.96E-07	-0.62	7.14E-15	-0.03	0.717	-0.018	0.887	0.406	0.004	0.093	0.343	0.274	0.006
Myristic.acid	-1.04	5.20E-14	-1.52	4.56E-23	-0.11	0.381	0.127	0.480	0.366	0.051	0.207	0.229	0.575	0.001
Octanoic.acid	-0.04	6.33E-01	-0.08	2.84E-01	-0.21	0.007	0.059	0.538	-0.091	0.348	0.036	0.734	0.114	0.288

ESM Table 2. Beta coefficients of metabolites and their P values from the mixed effect model with experiment, time and the relative interaction as within factor and glycaemic status as between factor

Metabolite	time2	Pvalue	time3	Pvalue	IGIVI	Pvalue	IGT	Pvalue	T2DM	Pvalue	time2:IGIVI	Pvalue	time3:IGIVI	Pvalue
Heptadecanoic.acid.d33	537.63	3.38E-16	401.24	9.70E-11	-99.0	0.08	95.7	0.39	106.3	0.34	-377.7	5.81E-06	-285.1	0.000
Glutamic.acid.d5	548.56	9.87E-16	413.37	1.47E-10	-100.2	0.09	97.0	0.40	106.6	0.35	-383.8	9.48E-06	-294.5	0.001
Succinic.d4.acid	556.13	3.92E-16	414.58	1.12E-10	-102.2	0.08	97.9	0.39	108.3	0.35	-390.0	6.36E-06	-293.3	0.001
Valine.d8	579.35	9.64E-16	436.60	1.44E-10	-105.7	0.09	102.5	0.40	111.3	0.36	-407.1	8.66E-06	-310.5	0.001
C11	669.62	2.45E-16	495.30	1.06E-10	-122.9	0.08	118.3	0.39	131.1	0.34	-471.7	4.87E-06	-351.0	0.001
C15	657.68	3.58E-16	491.86	9.37E-11	-122.0	0.08	117.6	0.38	130.2	0.34	-462.0	5.96E-06	-349.0	0.000
C17	661.61	2.81E-16	493.04	8.97E-11	-122.1	0.08	118.7	0.38	130.8	0.34	-464.8	5.42E-06	-349.7	0.000
C21	657.53	3.19E-16	491.15	9.01E-11	-121.5	0.08	117.0	0.39	129.6	0.34	-462.1	5.65E-06	-348.8	0.000
C25	653.85	3.77E-16	489.40	9.44E-11	-121.6	0.08	114.9	0.39	127.8	0.34	-459.5	6.04E-06	-347.5	0.000
X3.Hydroxybutyric.acid	169.05	1.13E-13	94.04	7.01E-06	-52.0	0.01	38.2	0.39	60.5	0.18	-129.5	1.16E-05	-76.9	0.007
Alanine	222.56	2.90E-16	163.95	1.38E-10	-36.1	0.12	47.6	0.29	50.1	0.27	-157.5	4.72E-06	-119.6	0.000
Arachidonic.acid	265.71	3.25E-16	196.40	1.33E-10	-51.4	0.07	53.1	0.33	59.3	0.28	-187.5	5.27E-06	-141.7	0.000
Citric.acid	230.11	7.25E-16	163.36	8.94E-10	-45.7	0.06	45.9	0.34	58.7	0.23	-164.4	5.63E-06	-118.7	0.001
Glutamic.acid	185.48	6.39E-16	130.77	1.03E-09	-33.5	0.09	23.8	0.56	41.6	0.32	-133.3	4.75E-06	-95.7	0.001
Glycine	177.48	8.46E-16	134.07	1.20E-10	-32.4	0.09	30.8	0.41	31.6	0.40	-123.4	1.01E-05	-93.7	0.001
Isoleucine	173.32	1.18E-14	114.61	4.15E-08	-37.3	0.06	32.7	0.40	43.4	0.27	-124.8	1.45E-05	-83.4	0.003
Leucine	201.11	9.19E-15	137.27	1.48E-08	-42.8	0.06	35.6	0.43	50.9	0.26	-144.5	1.36E-05	-100.0	0.002
Linoleic.acid	296.00	1.38E-15	204.00	3.44E-09	-62.0	0.05	59.1	0.37	70.1	0.29	-212.8	6.40E-06	-150.2	0.001
Malic.acid	144.16	1.11E-15	101.11	1.75E-09	-27.6	0.08	32.3	0.30	46.3	0.14	-107.3	2.96E-06	-79.0	0.000
Methionine	155.52	4.90E-15	104.91	1.42E-08	-33.4	0.05	32.4	0.34	41.8	0.22	-107.4	2.19E-05	-74.4	0.003
Palmitic.Acid	325.24	5.54E-16	228.00	1.13E-09	-65.8	0.06	64.7	0.36	71.2	0.31	-232.3	5.12E-06	-164.3	0.001
Phenylalanine	182.08	7.35E-16	127.07	1.56E-09	-36.7	0.06	34.4	0.38	42.5	0.28	-128.8	6.90E-06	-90.4	0.001
Proline	218.16	6.14E-15	157.12	2.30E-09	-41.5	0.09	40.8	0.39	42.1	0.38	-154.7	1.49E-05	-113.8	0.001
Serine	190.52	3.59E-15	131.75	5.66E-09	-36.8	0.08	35.8	0.39	35.2	0.40	-134.4	1.34E-05	-92.9	0.002
Stearic.acid	297.80	7.96E-16	211.31	9.66E-10	-60.3	0.06	56.3	0.38	62.2	0.33	-211.4	6.61E-06	-152.0	0.001
Threonine	200.45	8.73E-16	142.11	1.06E-09	-39.8	0.06	42.3	0.31	40.5	0.33	-141.6	7.54E-06	-101.9	0.001
Tyrosine	358.79	1.00E-15	257.56	7.69E-10	-64.6	0.09	61.9	0.41	80.3	0.29	-254.7	7.23E-06	-183.4	0.001
Valine	216.08	6.75E-16	154.29	7.06E-10	-42.7	0.06	38.4	0.40	49.6	0.28	-153.8	5.90E-06	-111.0	0.001
alpha.Tocopherol	472.05	4.12E-16	351.42	1.22E-10	-86.1	0.08	90.5	0.35	104.5	0.28	-332.3	6.02E-06	-249.5	0.001
Oleic.acid	396.33	9.91E-16	265.09	6.96E-09	-84.0	0.05	80.2	0.37	89.6	0.31	-284.0	6.03E-06	-191.6	0.002
Succinic.acid	450.75	3.17E-16	332.95	1.34E-10	-83.6	0.08	74.6	0.42	97.3	0.30	-318.7	5.02E-06	-236.1	0.001
Fumaric.acid	465.46	9.76E-16	349.36	1.67E-10	-85.9	0.09	80.7	0.41	95.8	0.33	-327.2	8.67E-06	-246.4	0.001
X3.4.Dihydroxybutanoic.acid	378.75	8.15E-16	279.27	2.73E-10	-73.1	0.07	66.9	0.40	81.0	0.31	-268.6	6.84E-06	-201.9	0.001
Aspartic.acid	444.63	2.32E-15	334.41	2.87E-10	-80.9	0.10	74.6	0.43	88.2	0.36	-313.7	1.12E-05	-241.2	0.001
X2.4.Dihydroxybutyric.acid	375.95	9.85E-16	272.99	5.21E-10	-73.1	0.07	67.9	0.39	86.1	0.28	-266.6	7.34E-06	-198.0	0.001
Tryptophan	502.46	3.00E-16	373.45	1.03E-10	-88.2	0.09	81.7	0.43	100.4	0.34	-354.6	5.09E-06	-266.8	0.000
Lactic.Acid	641.88	2.97E-16	471.43	1.56E-10	-116.2	0.08	117.1	0.38	131.4	0.32	-454.5	4.73E-06	-340.2	0.000
Glyceric.acid	482.62	3.92E-16	351.65	2.48E-10	-91.4	0.07	88.6	0.38	104.0	0.30	-342.4	5.09E-06	-253.0	0.001

Pyruvic.acid	578.81	2.09E-16	431.47	7.21E-11	-98.5	0.10	105.8	0.37	111.4	0.35	-408.6	4.38E-06	-310.0	0.000
X2.Hydroxybutyric.acid	576.79	3.91E-16	409.96	5.74E-10	-109.9	0.07	107.9	0.37	127.5	0.29	-408.6	5.23E-06	-294.6	0.001
Cholesterol	657.92	3.57E-16	493.01	8.71E-11	-120.1	0.08	119.9	0.38	131.3	0.33	-462.6	5.84E-06	-350.1	0.000
X1.Dodecanol	514.72	2.70E-16	379.88	1.23E-10	-93.1	0.08	92.4	0.38	106.2	0.32	-363.4	4.83E-06	-271.4	0.000
X2.Aminobutyric.acid	571.28	9.50E-16	417.99	3.94E-10	-103.8	0.09	101.0	0.40	113.0	0.34	-403.5	7.83E-06	-298.2	0.001
X2.Oxoisovaleric.acid	583.34	5.07E-16	428.60	2.22E-10	-107.7	0.08	104.9	0.39	122.2	0.32	-412.0	6.13E-06	-305.1	0.001
X2.Palmitoylglycerol	518.17	3.37E-16	383.44	1.31E-10	-97.5	0.07	96.6	0.36	101.7	0.34	-366.5	5.10E-06	-272.2	0.001
X3.Hydroxyisovaleric.acid	527.72	6.34E-16	389.60	2.20E-10	-100.1	0.08	98.8	0.37	109.1	0.33	-379.9	4.59E-06	-271.7	0.001
X3.Indoleacetic.acid	511.34	8.13E-16	380.16	2.05E-10	-98.2	0.07	69.6	0.51	121.0	0.25	-360.6	7.59E-06	-269.4	0.001
X3.methyl.oxo.pentanoic.acid	591.57	6.83E-16	424.58	6.01E-10	-114.0	0.07	108.8	0.38	133.2	0.29	-416.4	7.35E-06	-301.1	0.001
X4.Deoxytetronic.acid_1	513.73	1.29E-15	380.11	3.33E-10	-93.8	0.09	96.1	0.38	100.6	0.36	-363.2	8.64E-06	-273.1	0.001
X4.Hydroxybutanoic.acid	486.73	3.19E-16	359.52	1.34E-10	-94.4	0.07	88.2	0.38	99.6	0.32	-345.1	4.74E-06	-255.4	0.001
X4.Hydroxyphenyllactic.acid	515.56	4.77E-16	378.53	2.19E-10	-96.3	0.08	92.5	0.38	102.4	0.34	-365.2	5.67E-06	-272.5	0.001
X9.Hexadecenoic.acid_1	550.01	1.35E-15	379.30	3.34E-09	-103.4	0.08	111.0	0.36	104.1	0.39	-396.2	6.15E-06	-279.0	0.001
Aminomalonic.acid	617.83	3.53E-16	458.99	1.17E-10	-114.3	0.08	109.8	0.39	118.2	0.35	-435.8	5.47E-06	-327.1	0.001
Asparagine	542.49	5.48E-16	395.26	3.12E-10	-98.0	0.09	100.0	0.38	110.5	0.33	-382.2	6.64E-06	-280.5	0.001
Creatinine	615.33	4.22E-16	461.59	9.43E-11	-114.1	0.08	102.3	0.42	115.6	0.36	-434.5	5.72E-06	-328.0	0.000
Decanoic.acid	553.49	2.81E-16	405.50	1.64E-10	-109.6	0.06	101.9	0.38	104.8	0.36	-389.0	5.37E-06	-285.9	0.001
Diethylene.glycol	562.16	3.76E-16	421.28	9.02E-11	-101.4	0.09	101.5	0.39	112.7	0.34	-394.6	6.17E-06	-299.8	0.000
Docosahexaenoic.acid	565.71	6.16E-16	417.19	2.24E-10	-108.8	0.07	110.8	0.35	118.5	0.31	-396.9	7.51E-06	-298.1	0.001
Dodecanoic.acid	531.38	1.44E-15	384.60	7.47E-10	-106.4	0.06	98.8	0.39	114.8	0.32	-373.6	9.99E-06	-276.7	0.001
Glycerol_1	729.82	6.04E-17	539.85	3.99E-11	-134.9	0.07	131.6	0.38	147.7	0.32	-506.1	3.88E-06	-372.8	0.001
Glycerol.3.phosphate	602.25	3.75E-16	441.33	1.98E-10	-114.1	0.07	114.8	0.35	118.0	0.34	-424.5	5.67E-06	-315.6	0.001
Ketoleucine	599.47	7.52E-16	433.96	4.81E-10	-114.5	0.07	110.6	0.38	133.6	0.29	-423.5	7.10E-06	-308.9	0.001
Myo.Inositol	673.23	2.22E-16	504.69	6.13E-11	-123.1	0.08	120.7	0.38	134.8	0.33	-476.0	4.35E-06	-359.6	0.000
Myristic.acid	554.93	8.66E-16	384.42	2.20E-09	-115.7	0.05	110.0	0.36	125.9	0.30	-397.8	5.67E-06	-277.5	0.001
Octanoic.acid	596.91	2.44E-16	442.74	9.60E-11	-116.3	0.06	109.3	0.37	111.8	0.36	-419.9	5.00E-06	-312.4	0.001
Pentadecanoic.acid_1	504.75	1.50E-15	357.72	1.52E-09	-103.5	0.06	101.7	0.35	96.3	0.38	-363.7	6.36E-06	-259.4	0.001
Pyroglutamic.acid_1	557.12	8.08E-16	410.22	2.84E-10	-106.3	0.07	105.9	0.37	113.4	0.34	-393.0	7.53E-06	-291.5	0.001
Ribonic.acid_1	520.38	2.82E-16	382.43	1.47E-10	-98.1	0.07	98.3	0.34	106.0	0.30	-369.1	4.49E-06	-273.2	0.001
Terephthalic.acid	445.49	5.38E-16	334.86	1.04E-10	-82.8	0.08	83.0	0.37	92.1	0.33	-313.3	6.81E-06	-235.7	0.001
Triethylene.glycol	623.01	3.40E-16	465.23	9.54E-11	-115.5	0.08	109.8	0.39	123.4	0.34	-438.0	5.76E-06	-329.7	0.000
Uridine	535.78	3.56E-16	395.29	1.51E-10	-99.3	0.08	95.2	0.39	109.7	0.33	-376.3	5.97E-06	-278.1	0.001

ESM Table 3. Beta coefficients of metabolites with their P values from the mixed effect model multiplied by insulin with experiment, time and the relative interaction as within factor and glycaemic status as between factor

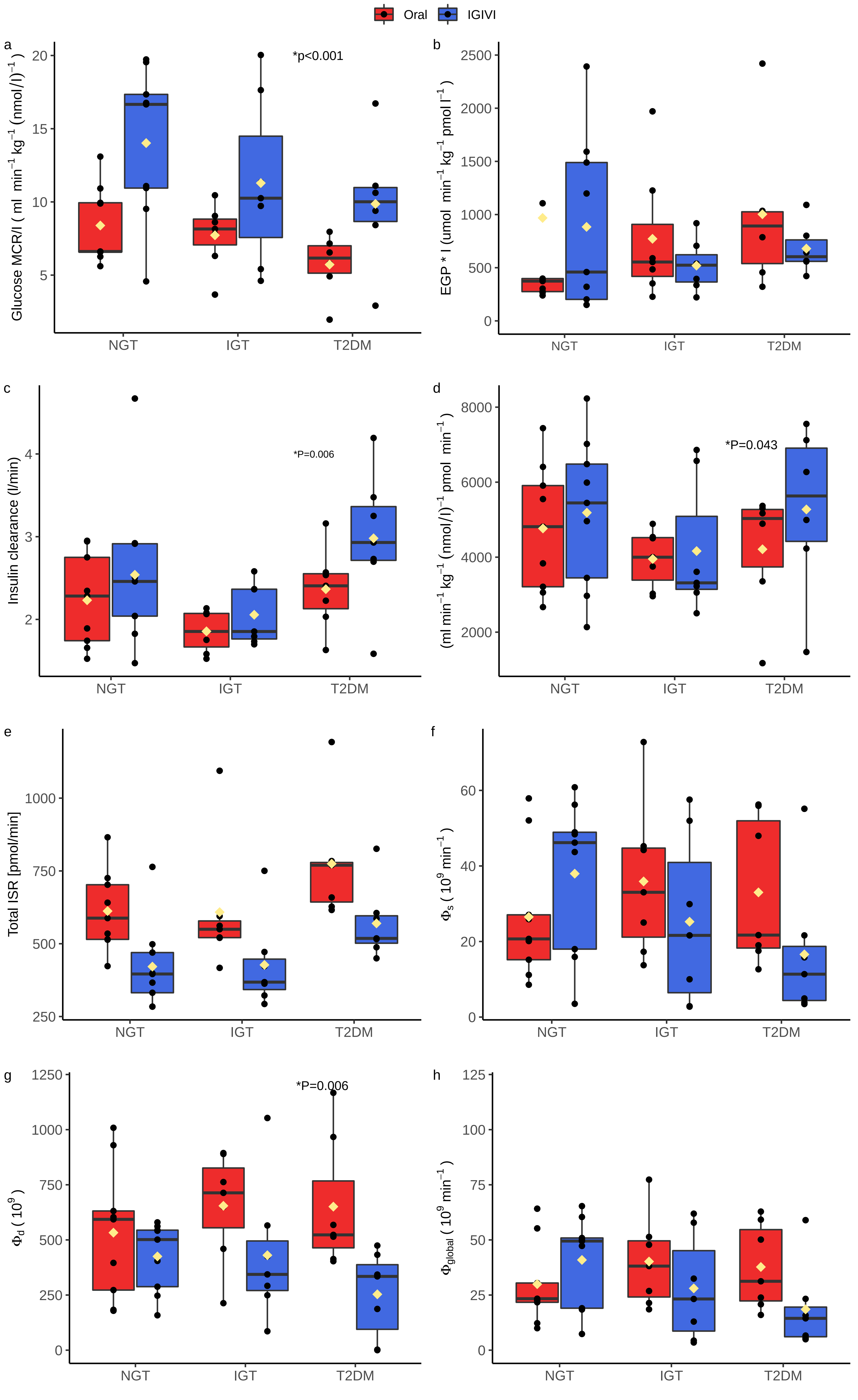

Oral
IGIVI
a
Glucose MCR/I (ml min^{-1} kg^{-1} (nmol/l)$^{-1}$)
*p<0.001
NGT
IGT
T2DM
b
EGP * I (umol min^{-1} kg^{-1} pmol l^{-1})
c
Insulin clearance (l/min)
*P=0.006
d
(ml min^{-1} kg^{-1} (nmol/l)$^{-1}$ pmol min^{-1})
*P=0.043
e
Total ISR [pmol/min]
f
Φ_s (10^9 min^{-1})
g
Φ_d (10^9)
*P=0.006
h
Φ_{global} (10^9 min^{-1})

ESM Figure 1

Whole-body insulin sensitivity measured with stable isotopes as insulin-mediated glucose metabolic clearance rate (MCR), i.e. Rate of glucose disappearance (Rd) from general circulation normalized by circulating levels of glucose and insulin (Rd/G)/I in relation to the glycaemic states (NGT, IGT and T2DM) and route of glucose administration (Oral vs IGIVI) (panel a). EGP measured by stable isotopes and multiplied by circulating levels of insulin was used as an index of hepatic insulin resistance (panel b). Insulin clearance computed according to the formula ISR-AUC/insulin-AUC − V×(Insulin at final time − Insulin at initial time)/ insulin-AUC (panel c). Disposition Index (DI) was computed as the product of insulin-mediated MCR (panel d) and the total insulin secretion rate (ISR) (panel e), computed as the area under the curve of ISR. Panel e reports the average insulin secretion resulting from the total ISR AUC. Static (Φ_s) (panel f) and dynamic (Φ_d) (panel g) β-cell glucose sensitivity and global β-cell glucose sensitivity (Φ_{global}) (panel h), which results from the sum of the two components above.

Black circles represent single subject values whereas yellow diamonds represent mean values.

* P level refers to the comparison between Oral administration and IGIVI
